# HepAssis2^®^ bioartificial liver system in treating acute‐on‐chronic liver failure patients: Findings from a phase 1 randomised, open‐label clinical trial

**DOI:** 10.1002/ctm2.70620

**Published:** 2026-02-18

**Authors:** Zibiao Zhong, Wenjin Liang, Yong'an Dai, Chengbiao Xue, Dawei Zhou, Zhigao Xu, Ling Li, Li Pan, Chongxiang He, Xin Zhou, Wei Zhou, Lihua Zhou, Zhongzhong Liu, Zhiping Xia, Xiaoli Fan, Guizhu Peng, Yanfeng Wang, Ping Zhou, Shaojun Ye, Qifa Ye

**Affiliations:** ^1^ Zhongnan Hospital of Wuhan University, Transplant Center of Wuhan University, Institute of Hepatobiliary Diseases of Wuhan University, Hubei Provincial Clinical Research Center for Natural Polymer Biological Liver, Hubei Key Laboratory of Medical Technology on Transplantation, National Quality Control Center for Donated Organ Procurement Wuhan China; ^2^ Department of Organ Transplantation Tongji Hospital, Tongji Medical College, Huazhong University of Science and Technology Wuhan China

1

Dear Editor,

Acute‐on‐chronic liver failure (ACLF) carries a grave prognosis of clinical syndrome, with its 28‐day mortality reported to be 50%.^1^ In China, a high‐endemic region for hepatitis B virus (HBV), over 80% of ACLF cases are HBV related.^2^ Because of the persistent shortage of donor livers and limited access to timely transplantation, the effective bridging therapies to sustain patients and improve survival are urgently needed.^3^ Bioartificial liver (BAL) systems represent a promising solution by providing both detoxification and synthetic hepatic functions.^4^, ^5^ Our research constitutes the first phase 1 randomised, open‐label trial for patients with HBV‐ACLF who were treated with the HepAssis2^®^ BAL system. The system's design (Figure S1) centres on an optimised bioreactor containing the immortalised L‐02 human foetal hepatocyte line.^6^

Ethical approval for this phase 1 trial was granted by the Medical Ethics Committee of Zhongnan Hospital of Wuhan University (no. 2016003). This trial was also formally registered at the Chinese Clinical Trial Registry (ChiCTR2300075781), 40 patients diagnosed with HBV‐ACLF were recruited from Zhongnan Hospital of Wuhan University over 2 years (January 2018 to December 2019). The main inclusion criteria are as followed: (1) informed consent from patient/legal guardian; (2) the diagnosis of ACLF was applied according to the following criteria: serum total bilirubin >171 µmol/L (or daily increase ≥17.1 µmol/L) plus prothrombin activity ≤40%; and (3) none of end‐stage complications (irreversible respiratory failure, cerebral herniation, sepsis or other severe infections). Using a randomisation schedule generated with SPSS 17.0, the 40 subjects were randomly allocated to two groups in a sequential order of enrollment. One group (*N* = 17 following exclusions) was treated with the combination of plasma exchange and the dual plasma molecular adsorption system (PE + DPMAS) according to the clinical guidelines for ACLF, and the other received the HepAssis2^®^ BAL system therapy (*N* = 19 after exclusions) (Figure 1), alongside standard medical care. The primary outcome is the safety assessment, while the secondary outcomes are the model for end‐stage liver disease (MELD) score, total bilirubin level, coagulation function and annual survival rate.

**FIGURE 1:**
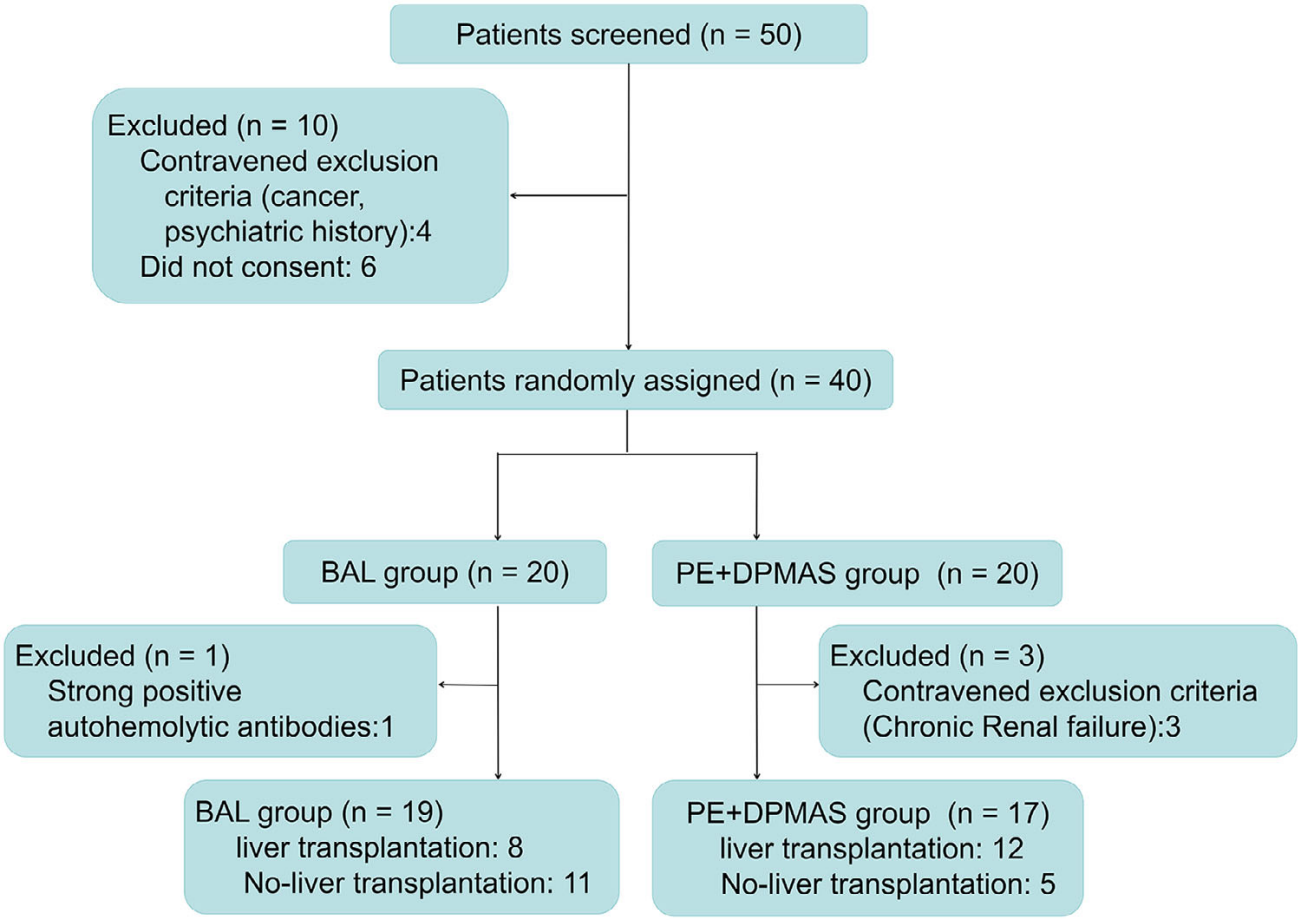
Flow chart of study selection.

Our key findings indicated that the BAL group received 1.5 ± .7 plasma‐free sessions, whereas the PE + DPMAS group received 1.4 ± .6 sessions, which require 2547.1 ± 451.5 mL of plasma per session (Table S1). Only three patients experienced chills during the initiation of BAL treatment, which can be resolved promptly with intravenous methylprednisolone. In addition, no other significant adverse events were observed, such as severe allergic reactions or embolic phenomena. Furthermore, we conducted a comparative analysis of laboratory indicators such as renal function, electrolytes and haematological parameters before and after treatment. The results showed no significant differences. It confirmed that the BAL treatment was safe (Figure 2).

**FIGURE 2:**
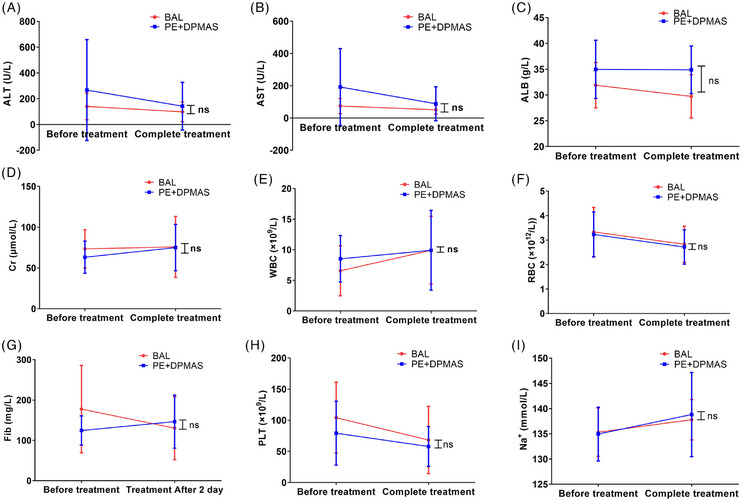
Comparative analysis of liver and kidney functions, blood cell counts, electrolytes and coagulation function indicators before and after treatment in two groups.

Compared to the control group, the BAL group demonstrated a superior efficacy in reducing MELD scores post‐treatment (*p* <.05), which implies a more substantial alleviation of hepatic disease severity. Additionally, reductions in total bilirubin and international normalised ratio (INR) during the first week post‐treatment were also more marked in the BAL group (Figure 3). We further applied the Kaplan‒Meier method for long‐term survival analysis and performed intergroup comparisons using the log‐rank test with Bonferroni correction. We have found that there are no statistically significant survival differences between the two groups at 28 days, 1 year or 5 years. Sequentially, a substantial number of patients underwent liver transplantation as a bridging procedure in two groups (12/17 in the control group vs. 8/19 in the BAL group). Further subgroup analysis revealed that among patients who did not undergo liver transplantation, the 1‐year survival rate was significantly higher in the BAL group compared to the PE + DPMAS group (*p* = .0312). In contrast, among the subgroup of patients who received liver transplantation, there was no statistically significant difference (*p* = .154) (Figure 4). These findings suggest that BAL therapy may confer a distinct survival advantage for ACLF patients who are not candidates for or do not receive liver transplantation.

**FIGURE 3:**
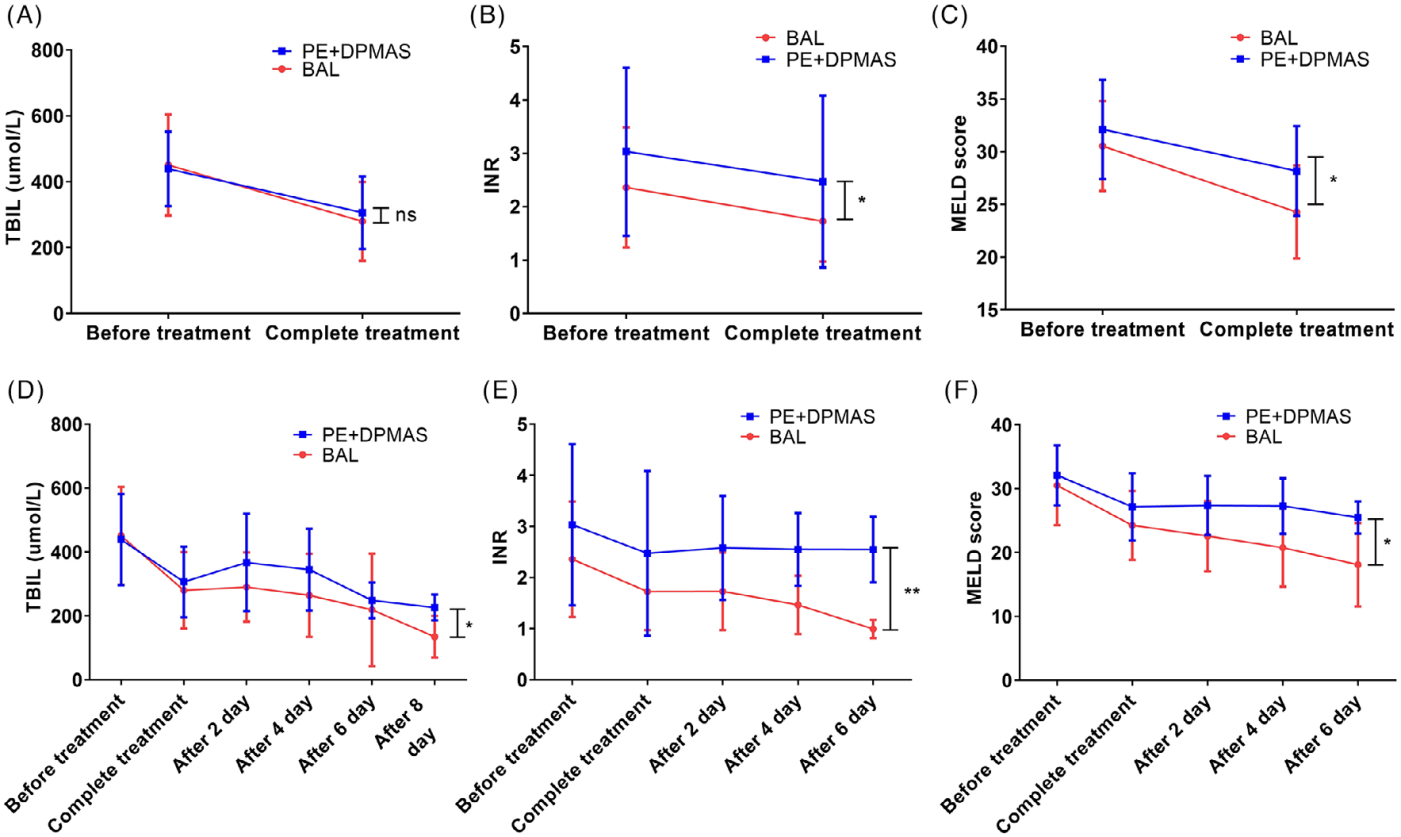
Comparative analysis of total bilirubin (TBIL), international normalised ratio (INR) and model for end‐stage liver disease (MELD) score before and after a single treatment (A‒C) and during the full‐cycle treatment (D‒F) in two groups.

**FIGURE 4:**
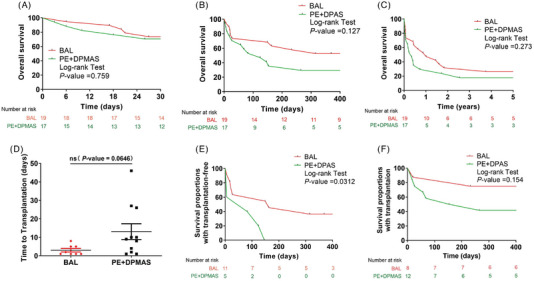
Comparison of the 28 days, 1 year and 5 years overall survival rates of patients of two groups (A‒C), the waiting time (D) for bridging liver transplantation, and with transplantation‐free (E) or with transplantation (F) in two groups.

In conclusion, this first‐in‐human clinical trial of BAL therapy for ACLF patients confirmed its comparable safety to the conventional PE + DPMAS regimen and highlighted its clinical advantage in improving the 1‐year overall survival rate for patients who did not undergo liver transplantation. These findings underscore its potential as a key bridging therapy for ACLF patients. Additionally, the plasma‐free session of BAL therapy further enhanced its safety profile. However, the main limitations of this trial included its single‐centre design and relatively limited number of participants. Furthermore, differences were observed in baseline prothrombin time, INR and alanine aminotransferase. These differences are likely attributable to substantial individual variability and the limited sample size; this can also be seen from the standard deviation data. Nevertheless, there was no significant difference in the key baseline assessment indicator of the MELD score. Furthermore, after correcting the laboratory parameters, our findings remained unchanged. With advances in multidisciplinary integration, BAL therapy stands at the forefront of liver regenerative medicine.^7^, ^8^ These advances provide a more effective and feasible alternative for the treatment of liver failure.^9^, ^10^ Nevertheless, larger‐scale, multicentre trials are warranted in the future to confirm these promising findings and further elucidate the role of BAL therapy.

## AUTHOR CONTRIBUTIONS

Yanfeng Wang, Ping Zhou, Shaojun Ye and Qifa Ye were responsible for the conception and supervision of the study. Zibiao Zhong and Wenjin Liang performed most of the experiments. Yong'an Dai, Chengbiao Xue, Dawei Zhou, Wei Zhou, Lihua Zhou, Zhongzhong Liu, Zhigao Xu, Xiaoli Fan, Guizhu Peng, Ling Li, Li Pan, Chongxiang He and Xin Zhou performed patient recruitment and clinical treatment. Zibiao Zhong and Wenjin Liang analysed the data and wrote the paper with suggestions from other authors.

## CONFLICT OF INTEREST STATEMENT

The authors declare they have no conflicts of interest.

## ETHICS STATEMENT

The studies involving human participants were reviewed and approved by the Ethics Committee of Zhongnan Hospital of Wuhan University (no. 2016003). All of the participants provided their written informed consent to participate in this study.

## Supporting information




**FIGURE S1**. Structural model diagram of HepAssis2^®^ bioartificial liver system.

**TABLE S1**. Characteristics of patients with Bioartificial liver (BAL) or combination of plasma exchange and the dual plasma molecular adsorption system (PE + DPMAS) group.

Supporting Information

## Data Availability

The datasets generated and analysed during this study are included in the published article and its Supporting Information. Further inquiries should be addressed to the corresponding authors.
