# Supplementary figures and images for: HepAssis2® bioartificial liver system in treating acute‐on‐chronic liver failure patients: Findings from a phase 1 randomised, open‐label clinical trial
Source: Clin Transl Med. 2026 Feb 18;16(2):e70620. doi: 10.1002/ctm2.70620 (PMC12914335; doi:10.1002/ctm2.70620)

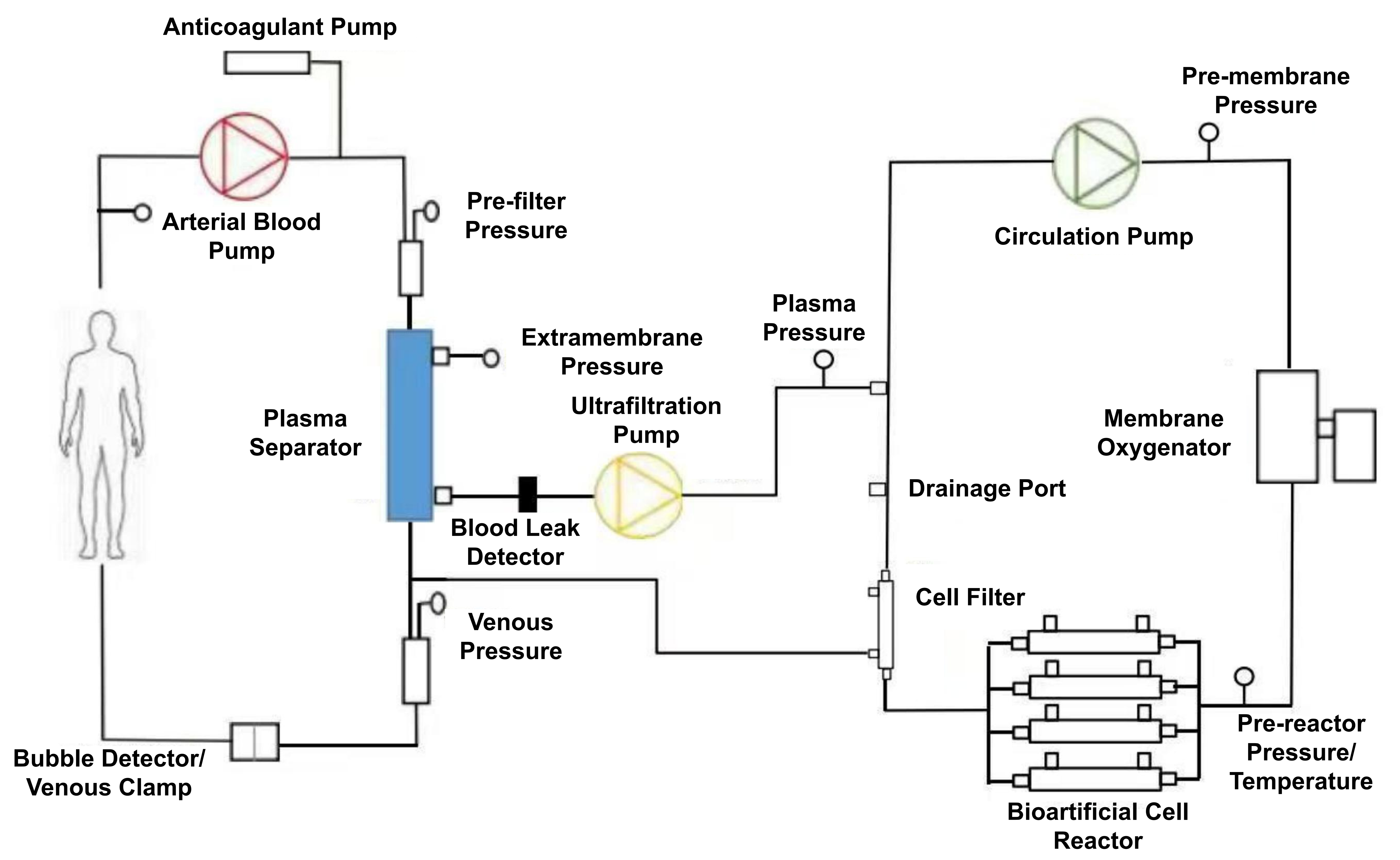

Supplement: Supplementary file 1 — FIGURE S1. Structural model diagram of HepAssis2® bioartificial liver system. [file CTM2-16-e70620-s002.png]
