# Supplementary material for: HepAssis2® bioartificial liver system in treating acute‐on‐chronic liver failure patients: Findings from a phase 1 randomised, open‐label clinical trial
Source: Clin Transl Med. 2026 Feb 18;16(2):e70620. doi: 10.1002/ctm2.70620 (PMC12914335; doi:10.1002/ctm2.70620)
Supplement: Supplementary file 2 — TABLE S1. Characteristics of patients with Bioartificial liver (BAL) or combination of plasma exchange and the dual plasma molecular adsorption system (PE + DPMAS) group. [file CTM2-16-e70620-s003.docx]

Table . Characteristics of patients with BAL or PE+DPMAS group.

| Patient Characteristic | BAL group (N=19) | PE+DPMAS group (N=17) | *P-*value |
| --- | --- | --- | --- |
| Age (yr) | 49.1±12.2 | 46.9±10.7 | 0.567 |
| Gender |  |  | 0.053 |
| Male | 16 | 14 |  |
| Female | 3 | 3 |  |
| Laboratory data |  |  |  |
| RBC (×10^12^/L) | 3.3±1.0 | 3.2±0.9 | 0.746 |
| WBC (×10^9^/L) | 6.6±4.1 | 8.5±3.8 | 0.148 |
| PLT (×10^9^/L) | 104.1±57.1 | 79.2±51.4 | 0.179 |
| Hb (g/L) | 106.7±25.9 | 107.1±24.4 | 0.961 |
| PT (s) | 25.7±11.2 | 36.7±18.9 | **0.038** |
| INR | 2.4±1.1 | 3.4±1.6 | **0.024** |
| Fib (mg/L) | 177.6±108.2 | 124.4±36.1 | 0.078 |
| ALB (g/L) | 31.9±4.4 | 35.0±5.6 | 0.074 |
| TB (µmol/L) | 451.1±153.7 | 439.1±143.0 | 0.810 |
| ALT (U/L) | 74.4±46.6 | 192.3±238.3 | **0.042** |
| AST (U/L) | 139.9±103 | 226.6±391.9 | 0.183 |
| Cr (µmol/L) | 73.4±23.4 | 63.1±19.7 | 0.165 |
| K^+^ (mmol/L) | 3.9±0.7 | 4.0±1.0 | 0.722 |
| Na^+^ (mmol/L) | 135.3±4.8 | 134.9±5.3 | 0.826 |
| MELD | 30.5±6.3 | 32.1±4.7 | 0.399 |
| Treatment frequency | 1.5±0.7 | 1.4±0.6 | 0.607 |
| Plasma usage (mL) | 0 | 2547.1±451.5 | **<0.0001** |

BAL: bioartificial liver; PE: plasma exchange; DPMAS: double plasma molecular adsorption system;ALB: albumin; TB: total bilirubin; ALT: alanine aminotransferase; AST: aspartate aminotransferase; Cr: creatinine; K+:potassium; Na+: sodium; Hb: hemoglobin; RBC: red blood cell; WBC: white blood cell; PLT: platelet count; PT: pro-thrombin time; Fib: fibrinogen; MELD: Model for End-stage Liver Disease.
