# Supplementary material for: HepAssis2® bioartificial liver system in treating acute‐on‐chronic liver failure patients: Findings from a phase 1 randomised, open‐label clinical trial
Source: Clin Transl Med. 2026 Feb 18;16(2):e70620. doi: 10.1002/ctm2.70620 (PMC12914335; doi:10.1002/ctm2.70620)

**Ethical Approval of Clinical Research Projects by Medical Ethics Committee of Zhongnan Hospital, Wuhan University**  (Ethical Approval Number [2016003])

**Project Name**: HepAssis2® Bioartificial Liver System in Treating Acute-on-chronic Liver Failure Patients

**Project Sponsor**: Wuhan Tong Gan Biological Technology Co,Ltd.

**Project Leader**: Prof. Qifa Ye.

**Departmer**: Institute of Hepatobiliary Diseases of Wuhan University.

**Review of documents**: See annex

**Review categories**: Initial review

**Modalities of review**: Review of the Conference

**Date of review**:2016-04-01

**Locations of review**: Conference Room 2, 13th floor, Zhongnan Hospital Ambulatory

**Attendees**: Actually, there were 10 people, avoid with 1 person, abstaining 0 person.

**Results of the vote**: Agreed 8 votes with the necessary amendments, Consent 1 vote, Reconskdered with necessary amendments 0 votes，disagree 0 vote, suspense or terminate approved trials 0 vote.

**Review observations**:

1. As a result of the review by this Ethacs committee, the review decides as follows:

Consent after necessary amendments review after necessary revisions.

2. Frequency of annual /periodic tracking review: 12 months; Validity period: 2 years.

3. The Ethics Committee has the right to change the frequency of ongoing reviews based on actual progress.

**The Chairperson or the Deputy Chairperson sign**: Yuanzhen Zhang

**Date**: 2016-04-05

Medical Ethics Committee of Zhongnan Hospital, Wuhan University (Ced: 216.4-)

**NOTE:**

1. Conduct clinical research in accordance with the CFDA / GCP principles and the Declaration of Helsinki and in accordance with the protocol approved by this Ethics Committee to protect the health and rights of the subjects.

2. All projects involving the export of human genetic resources need to submit an application to the China Human Genetic Resources Management Office and obtain approval before the project is implemented.

3. In the course of research, changes to relevant documents such as research protocols and informed consent must be reviewed by the Ethics Committee before they can be implemented.

4. Report on the progress of research projects is submitted at the frequency of annual/periodic followed-up reviews established by the Ethics Committee.

5. Severe adverse events or unanticipated adverse events that affect the risk-benefit ratio of research shall be reported to this Ethics Committee in a timely manner.

Statement of Ethics Committee: The Ethics Committee is composed and works in strict accordance with China GCP and related laws and regulations.

Ethics Committee Address: 169 Donghu Road, Wuchang District,Wuhan City, Hubei Province; Zip code: 430071; Tel:027-67812787.

**List of members and attendance of medical ethics committee at Zhongnan Hospital, Wuhan University**

Time:April 1, 2016 2:30 PM; Locatior Conterence Room 2, 13th Floor, Ophthalmalogy.

| **Full name** | **Gender** | **Profession** | **Work unit** | **Position of the Ethics Committee** | **Autograph** |
| --- | --- | --- | --- | --- | --- |
| Zhang  Yuanzhen | Female | Obstetrics and gynecology | Zhongnan Hospital of Wuhan University | Chairman | Zhang  Yuanzhen |
| Zhang  Junjan | Male | Medicopsychology | Zhongnan Hospital of Wuhan University | Committee  member | Zhang  Junjan |
| Wang Youwei | Male | Pharmacology | Zhongnan Hospital of Wuhan University | Committee  member | Wang Youwei |
| Huang Jianying | Female | Medical Management | Zhongnan Hospital of Wuhan University | Committee  member | Huang Jianying |
| Wu Xiaoyan | Female | Nephrology | Zhongnan Hospital of Wuhan University | Committee  member | Wu Xiaoyan |
| Wang Yanfeng | Male | Surgery | Zhongnan Hospital of Wuhan University | Committee  member | Avoid |
| Zhou Fuxiang | Male | Oncology | Zhongnan Hospital of Wuhan University | Committee  member | Zhou Fuxiang |
| Hu Hankun | Male | Pharmacology | Zhongnan Hospital of Wuhan University | Committee  member | Hu Hankun |
| Zhen Fang | Female | Clinical Laboratory Diagnostics | Zhongnan Hospital of Wuhan University | Committee  member | Zhen Fang |
| Shun Caihua | Male | Law | Beijing Zhong Lun  Law Firm (Wuhan) | Committee  member | Shun Caihua |

**The attached original Version are as following:**


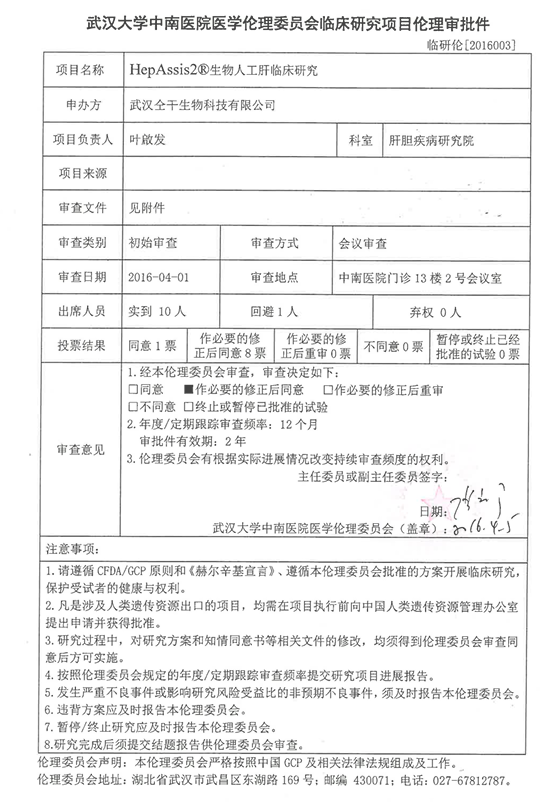

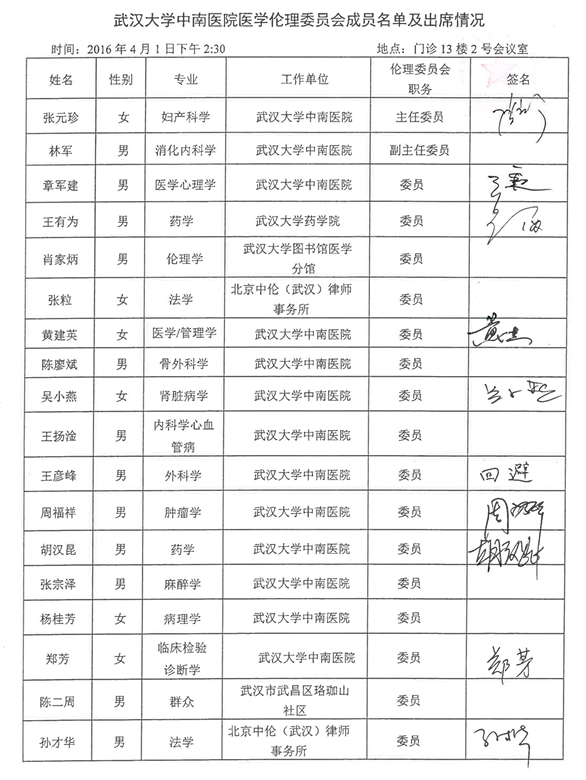

Supplement: Supplementary file 3 — Supporting Information [file CTM2-16-e70620-s001.docx]
